# Supplementary figures and images for: Soil type and fertilizer rate affect wheat (Triticum aestivum L.) yield, quality and nutrient use efficiency in Ayiba, northern Ethiopia
Source: PeerJ. 2022 May 9;10:e13344. doi: 10.7717/peerj.13344 (PMC9097669; doi:10.7717/peerj.13344)

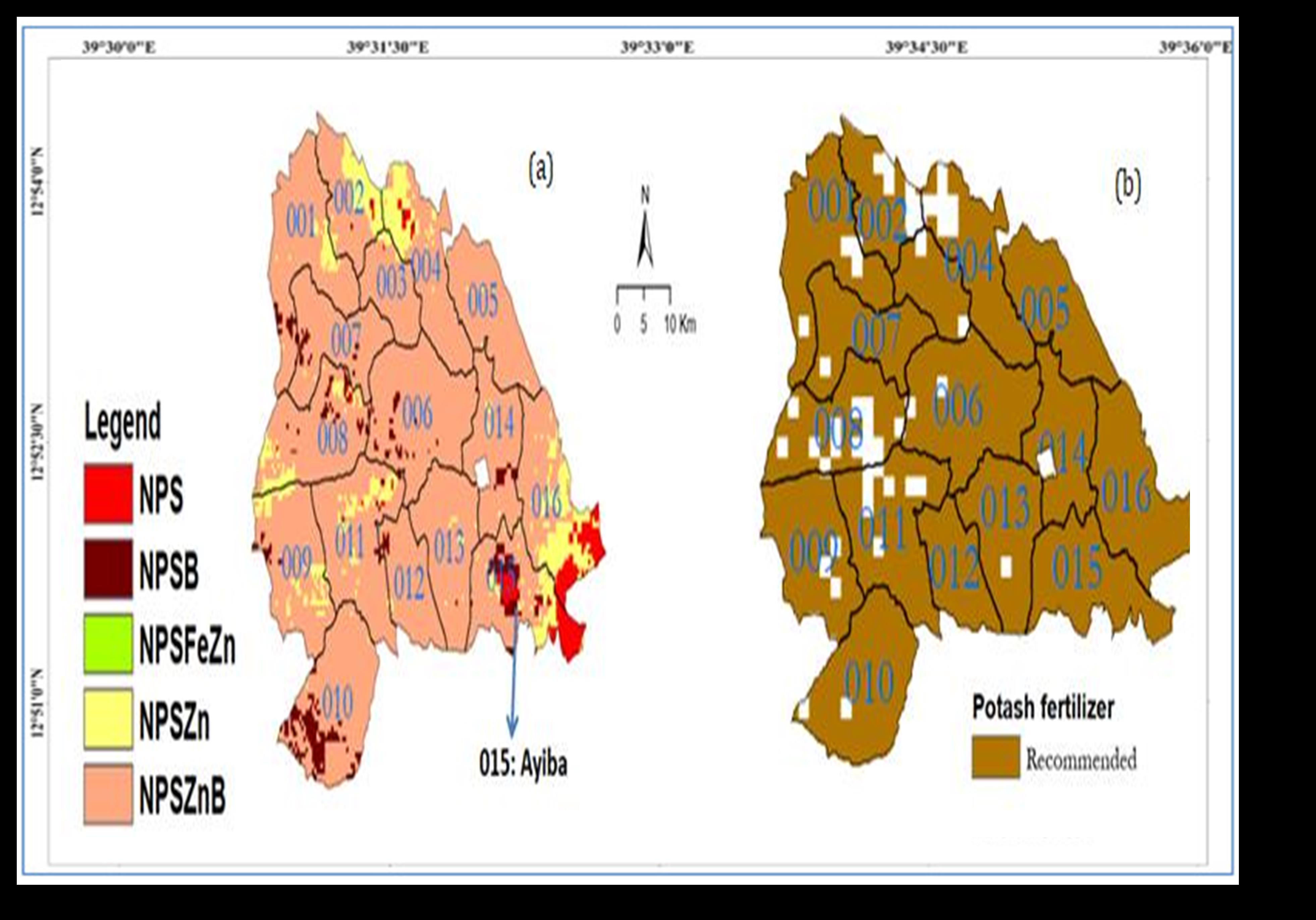

Supplement: Supplemental Information 2 — EthioSIS: Ethiopia Soil Information System [file peerj-10-13344-s002.png]

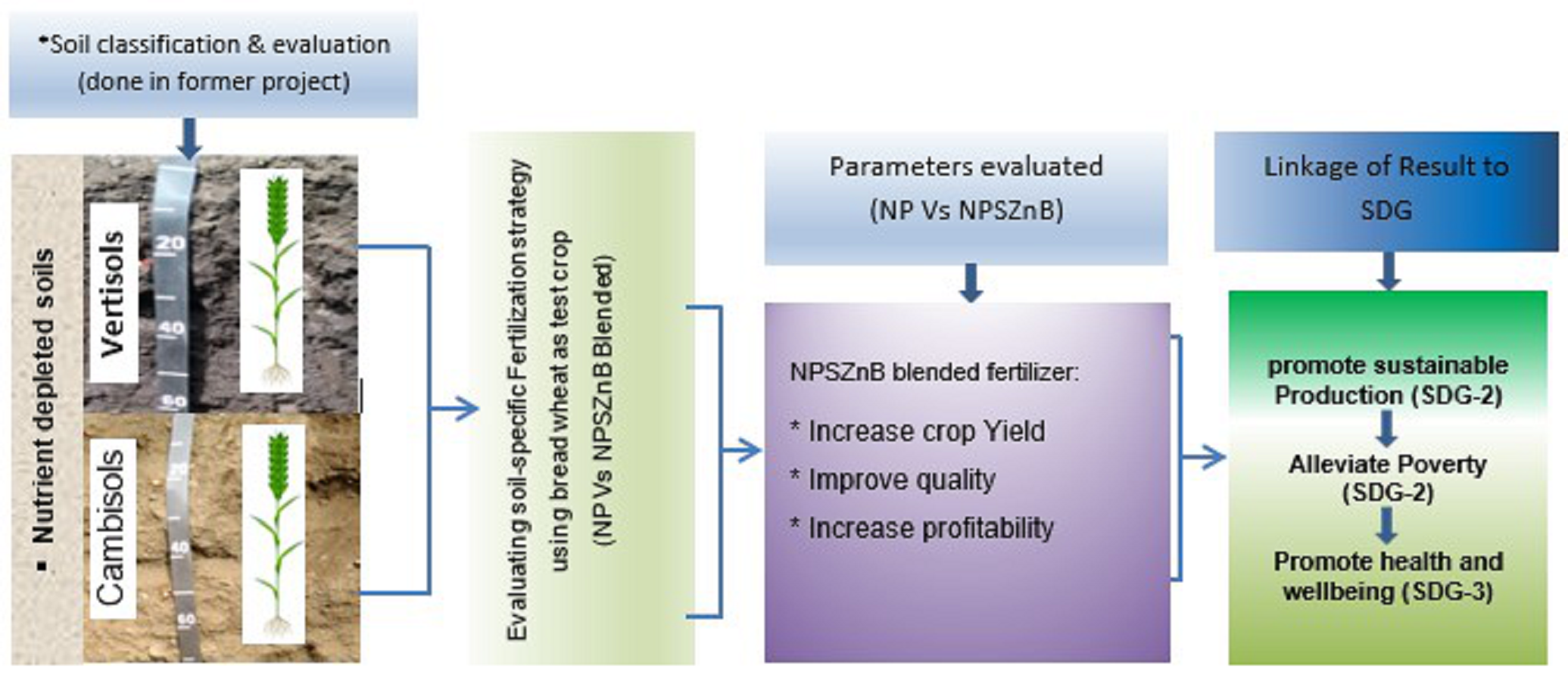

Supplement: Supplemental Information 3 [file peerj-10-13344-s003.png]
